# Supplementary material for: The impact of microRNAs on transcriptional heterogeneity and gene co-expression across single embryonic stem cells
Source: Nat Commun. 2017 Jan 19;8:14126. doi: 10.1038/ncomms14126 (PMC5253645; doi:10.1038/ncomms14126)
Supplement: Supplementary Information — Supplementary Figures [file ncomms14126-s8.pdf]

Supplementary Figure 1: Reintroduction of miRNAs into Dgcr8 knockout cells.

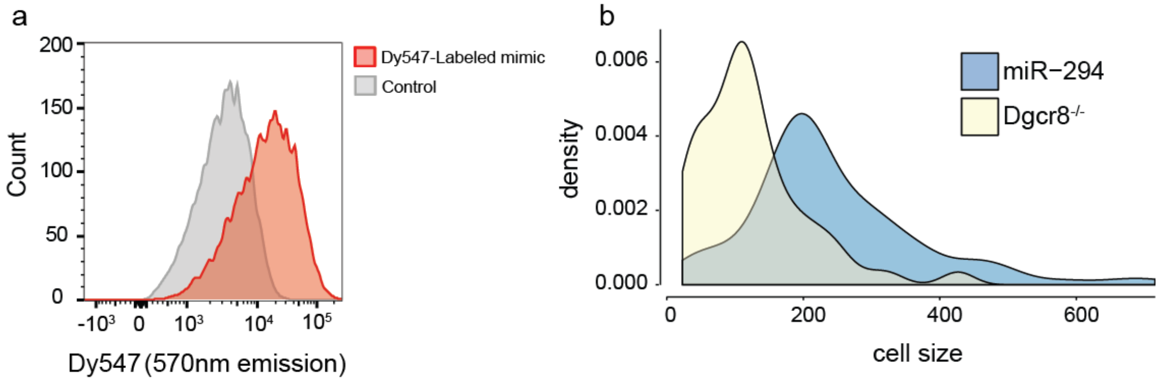

(**a**) Transfection efficiency: Fluorescent flow cytometry plot of cells transfected with Dy547 labelled mimic versus mock transfected. Note shift of entire population to the right on the fluorescence channel, consistent with transfection of most cells. (**b**) Cell size: Density maps of pixel counts of Dgcr8 knockout and miR-294 transfected cells.

Supplementary Figure 2: Sequencing counts.

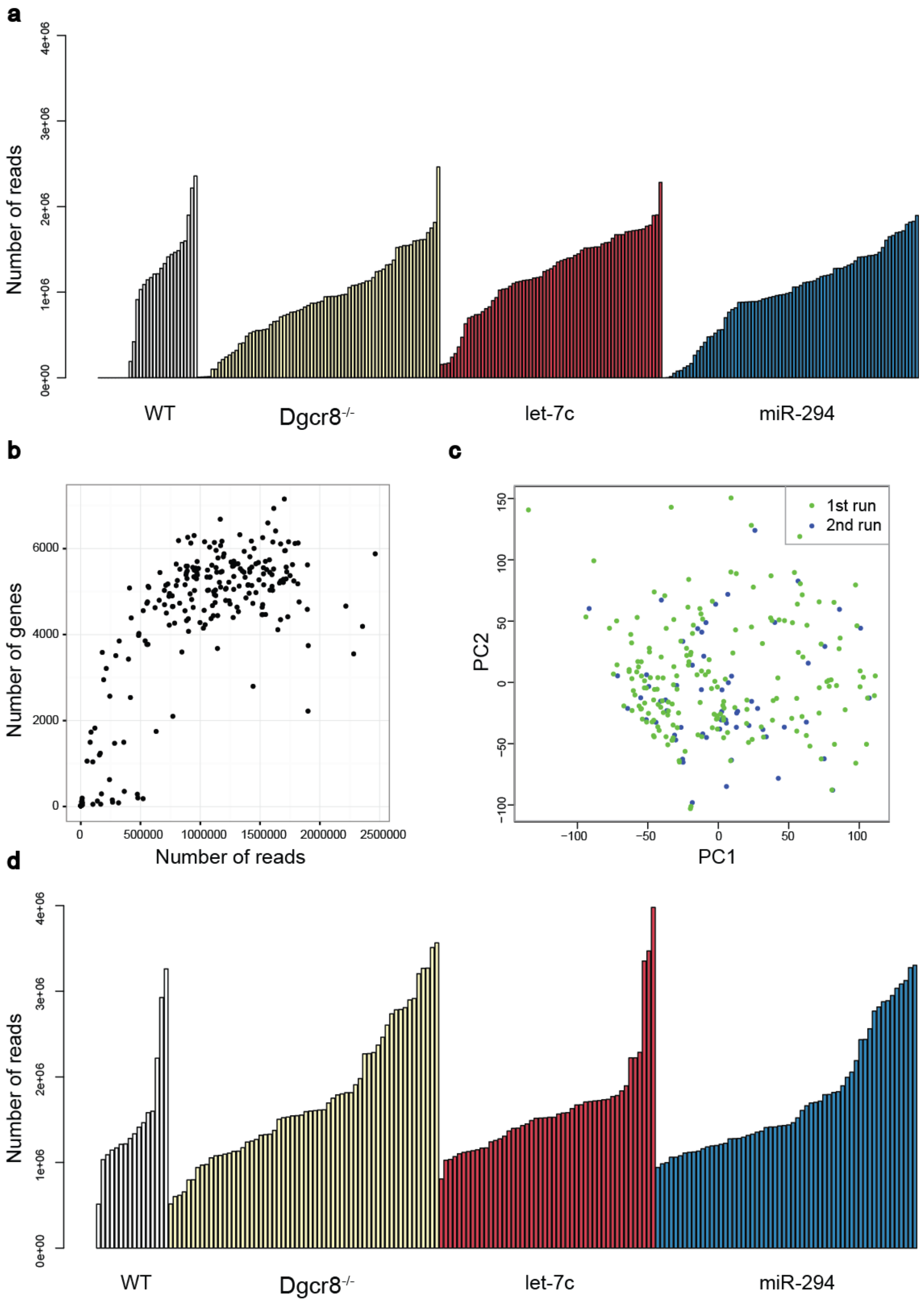

(**a**) Number of reads mapping to the genome for individual cells in each condition following first round of sequencing. (**b**) Number of genes with at least 10 counts in relation to number of reads. Note plateau starting at roughly 500,000 reads. (**c**) PCA analysis of samples run in first and second rounds of sequencing. (**d**) Number of total reads mapping genome following combination of first and second rounds.

Supplementary Figure 3: Read count relative to gene length.

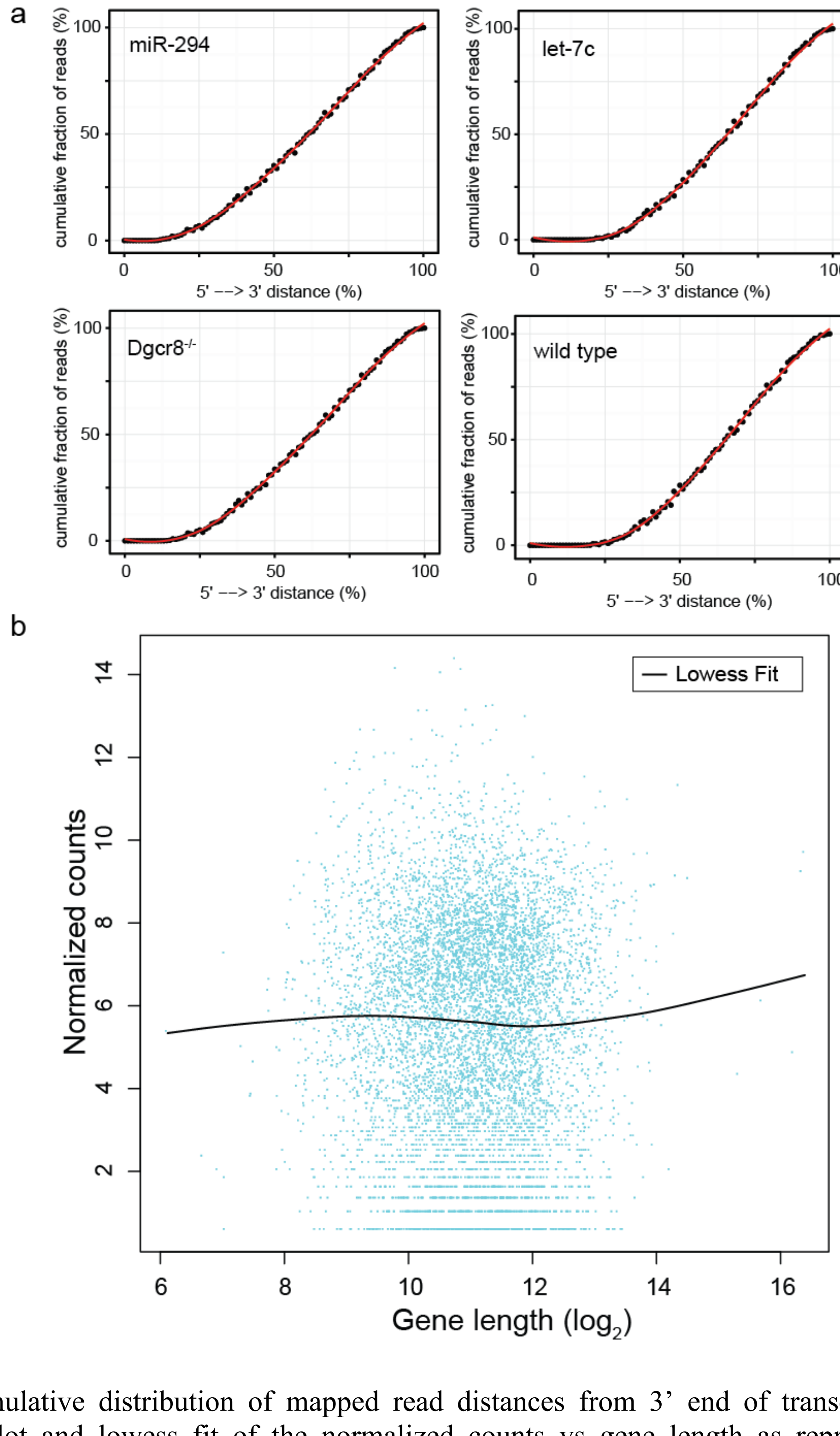

(**a**) Cumulative distribution of mapped read distances from 3’ end of transcripts. (**b**) Scatterplot and lowess fit of the normalized counts vs gene length as representative sample (Mock 11). Note lack of correlation between read count and gene length. Note, Mock was just our internal label for Dgcr8$^{-/-}$ cells.

Supplementary Figure 4: Sample Filters.

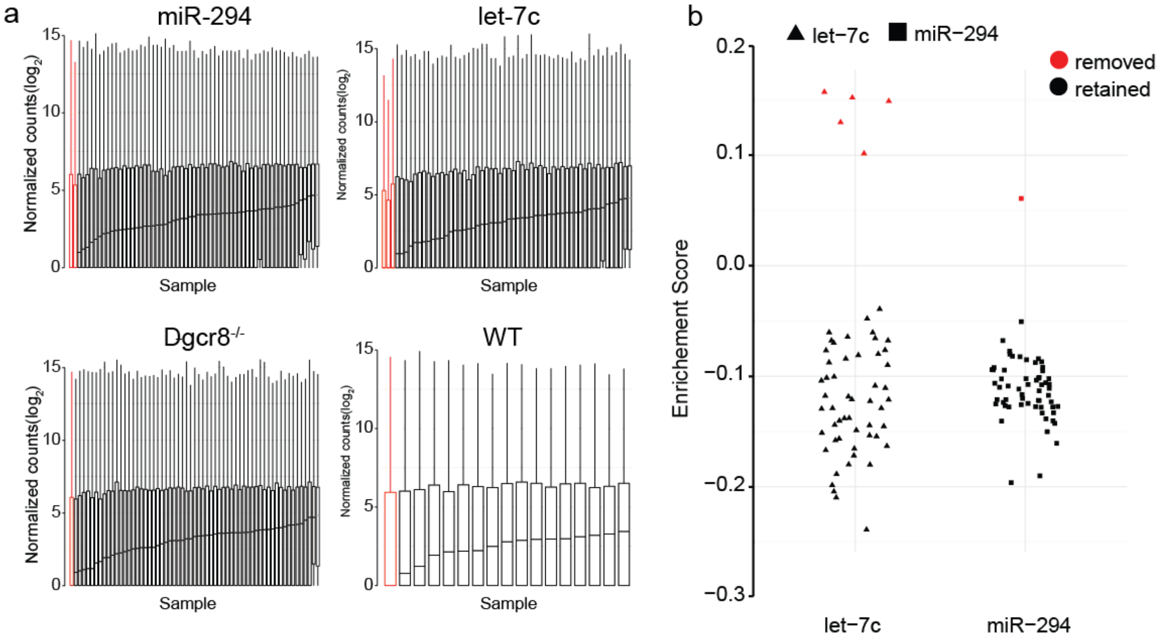

(**a**) Library diversity: Box plots of normalized counts per gene for each cell under each condition. Highlighted in red are samples that were removed due to low diversity (Median of 0) (**b**) Enrichment scores for predicted targets among differentially expressed genes (left, let-7c vs. mock; right, miR-294 vs. Dgcr8$^{-/-}$). Negative values of ES imply that used targets are down-regulated in the corresponding population of cells, while positive ES indicates opposite behavior. Highlighted in red are samples that were removed because the corresponding miRNA targets were not down-regulated as expected.

Supplementary Figure 5: Discriminatory pathways between miRNA transfected cells.

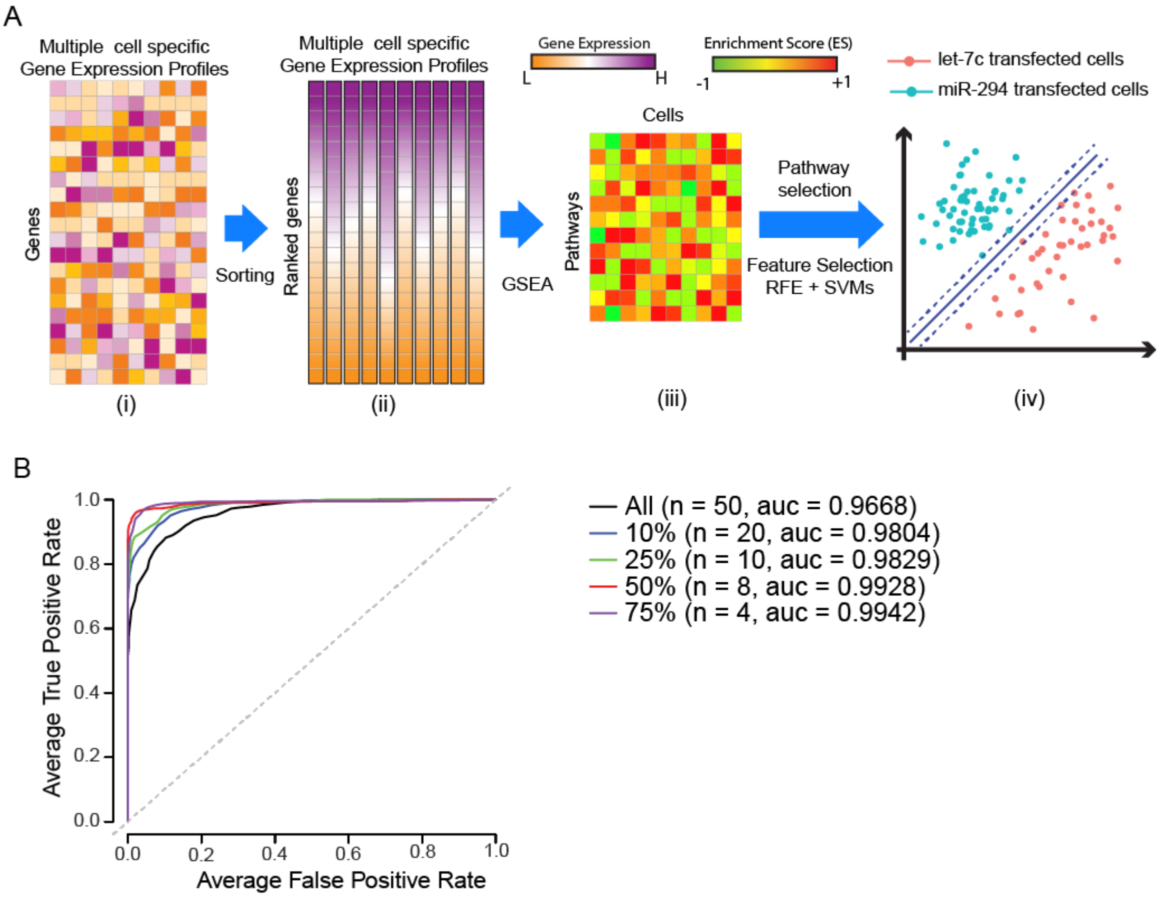

(**a**) Gene expression profiles (GEPs) for miR-294 and let-7c are first collected for each cell (i). GEPs are converted to ranks and individually sorted (ii). Gene expression ranks are converted to pathway Enrichment Scores (ES) trough Gene Set Enrichment Analysis (GSEA) (iii). RFE method is finally applied to select the most discriminatory pathways between miR-294 and let-7c transfected cells (iv). (**b**) Receiver operating characteristic (ROC) curve and corresponding area under the curve (AUC) as a function of different subsets of discriminant pathways defined using different cutoffs of relevance predicted by RFE algorithm. 10-fold cross-validation method was used for the estimation of the ROC curve.

Supplementary Figure 6: Cell-cell correlation density plots.

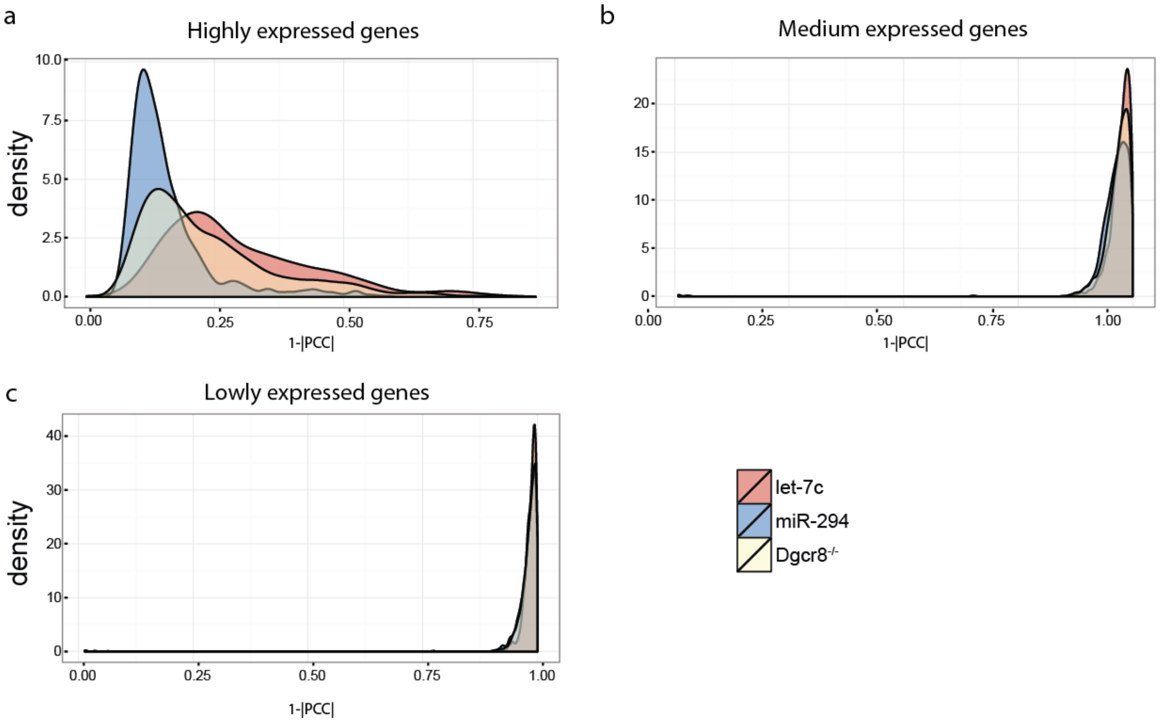

Similar to Fig. 3a (upper panel), but broken down into (**a**) highly, (**b**) intermediate, and (**c**) lowly expressed genes representing the top, middle, and bottom third of average expression values.

Supplementary Figure 7: let-7c and Dgcr8$^{-/-}$ subpopulations and PCA separation:

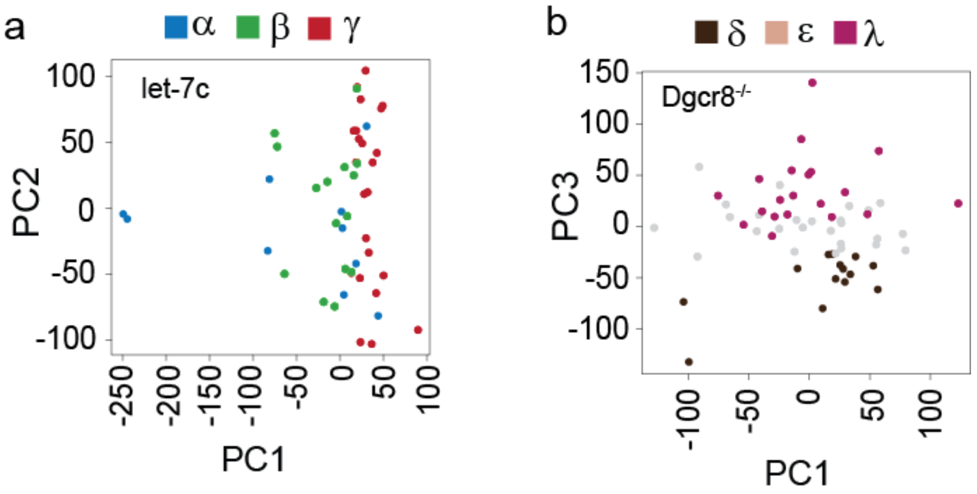

(**a**) PCA plot on let-7c cells shows a separation on the first principal component for the three cell subpopulations identified in the hierarchical clustering of Fig. 3b; while (**b**) PCA plot on Dgcr8$^{-/-}$ cells shows a separation on the third principal component.

Supplementary Figure 8: Identifying high confidence miRNA targets for RMI analysis.

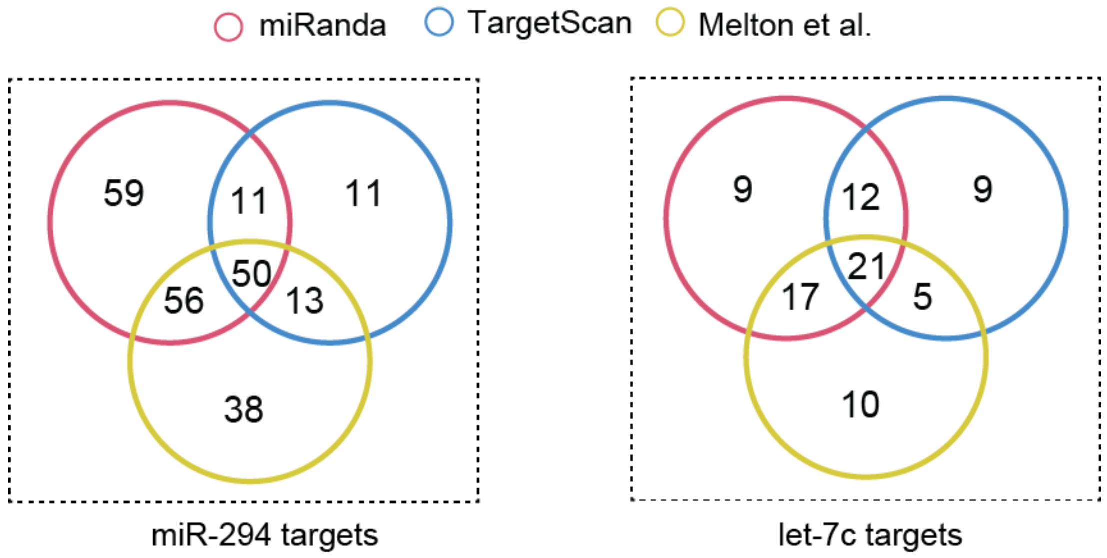

Venn diagram showing overlap of experimentally determined targets, miRanda-miRSVR computationally predicted targets and TargetScan computationally predicted targets (see Methods for details). Only the down-regulated genes in the population of individual cells receiving the corresponding miRNA (versus mock and adjusted p-value < 0.1) are showed. High confidence targets were defined as those predicted by at least two of these methods.

Supplementary Figure 9: Comparison between pair-wise and set-wise correlation approaches.

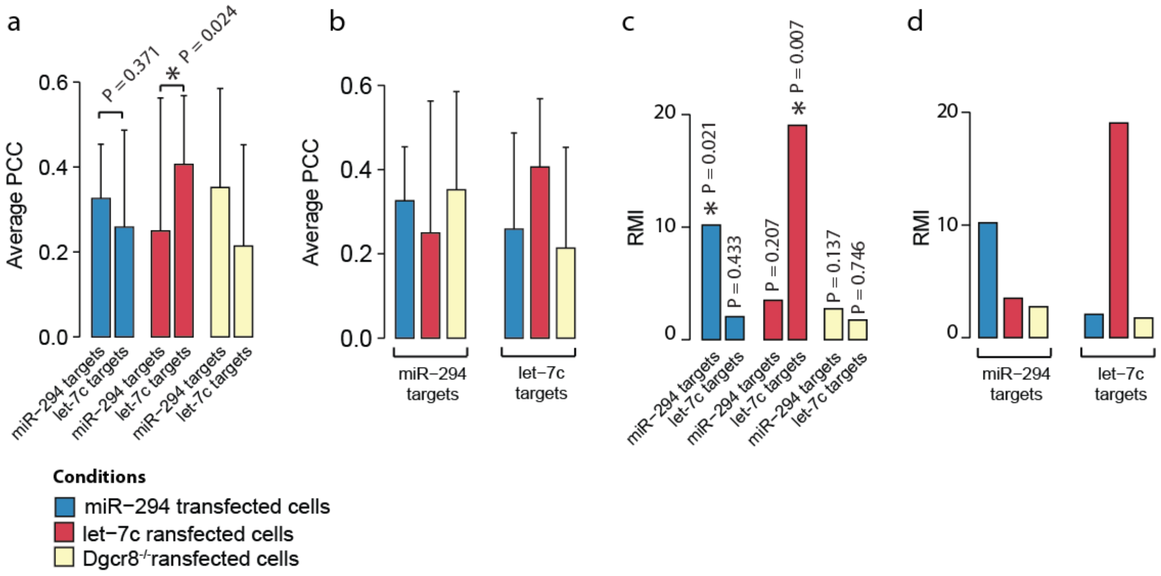

(**a**) Pearson Correlation Coefficient (PCC) between each pair of high confidence target genes both for miR-294 (n=33) and let-7c (n=41). The average correlation and its standard deviation for each set of target genes within each condition condition is shown. Only significant PPC values are considered (P>0.05). Asterisks report a significant difference between the two conditions (Mann–Whitney U test). (**b**) Same as (a) but the average correlation is compared across conditions. It can be appreciated that whereas an effect can still be detected when comparing miR-294 targets and let-7c targets within each condition, that is miR-294 targets are on average more correlated than let-7c target in miR-294 transfected cells, and vice-versa in let-7c transfected cells, the same is not true across conditions. (**c**) Rényi Multi-Information for a set 33 of high confidence miR294 targets or 41 let-7c high confidence targets is compared within conditions. P-values are report on top of each barplot. P-values were estimated with permutation test and are the same reported in Figure 4a-b. (**d**) Same as (b) but across conditions.

Supplementary Figure 10: Rényi Multi-Information distribution of miRNA targets across conditions.

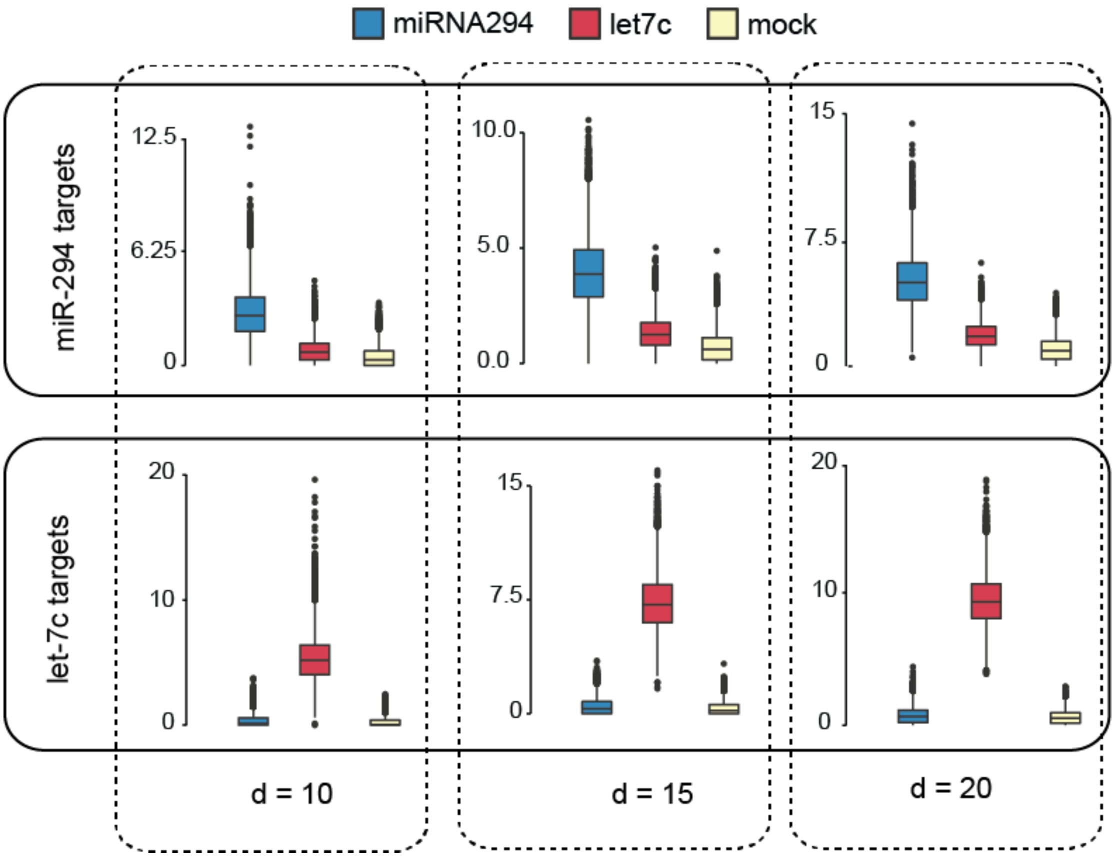

RMI distribution for each of the 3 conditions using larger sets of miRNA targets (see Methods). A distribution of RMIs was computed by randomly extracting different subset of *d* genes with replacement 10,000 times from the corresponding list of targets.

Supplementary Figure 11: Differential Rényi Multi-Information Pipeline.

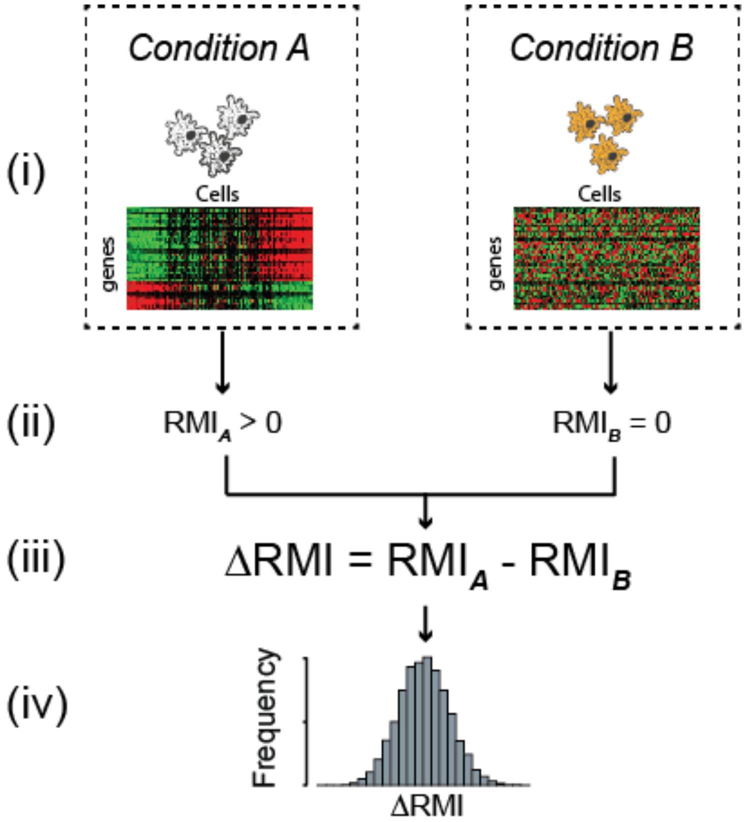

(i,ii) Given two conditions (*i.e.* populations of cells) A and B, and a set of d genes $G^1 \ldots G^d$, Rényi Multi-Information (RMI) among the d genes is individually estimated in each subset of GEPs. In this example the d genes are co-expressed only in the condition A (*i.e.* $RMI_A > 0$). (iii) ΔRMI is finally estimated as $RMI_A - RMI_B$. (iv) Significance is estimated via a permutation test as explained in the Materials and Methods section.

Supplementary Figure 12: Hallmark gene sets significantly modulated by either miR-294 or let-7c.

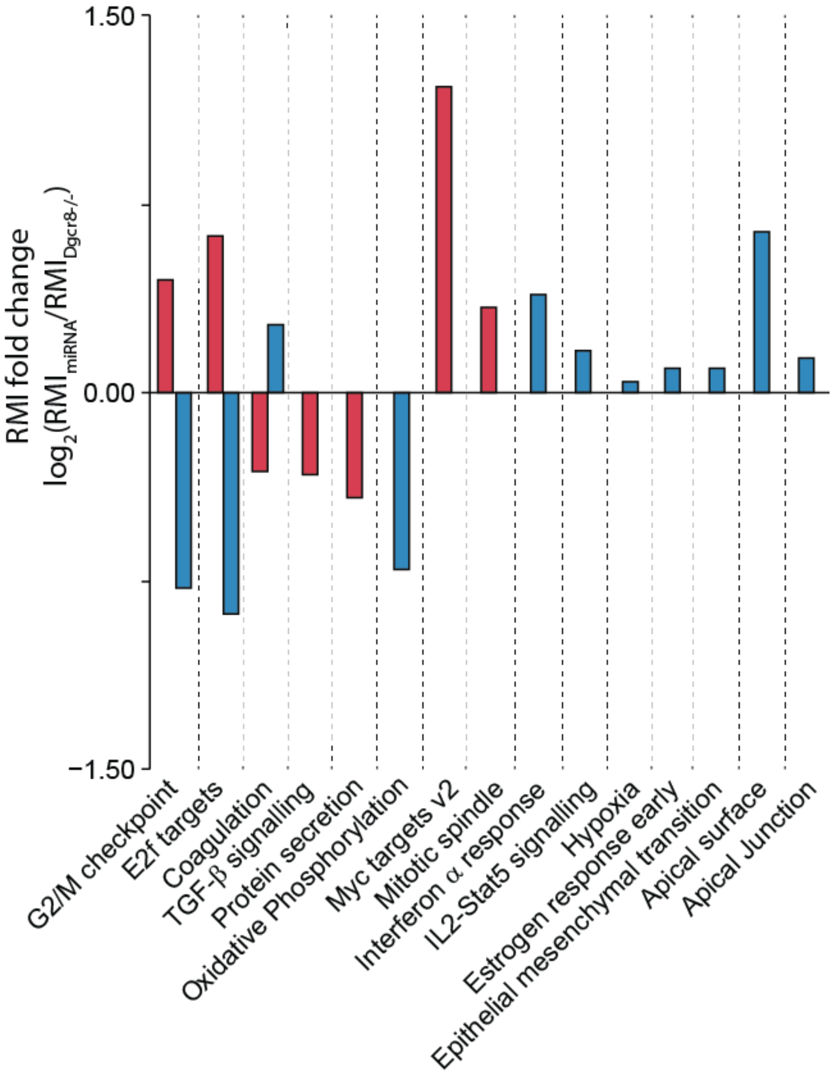

Same as Fig. 4f but without considering the miRNA targets reported in Table S5.

Supplementary Figure 13: miRNA targets overlap with MSigDb hallmarks gene sets:

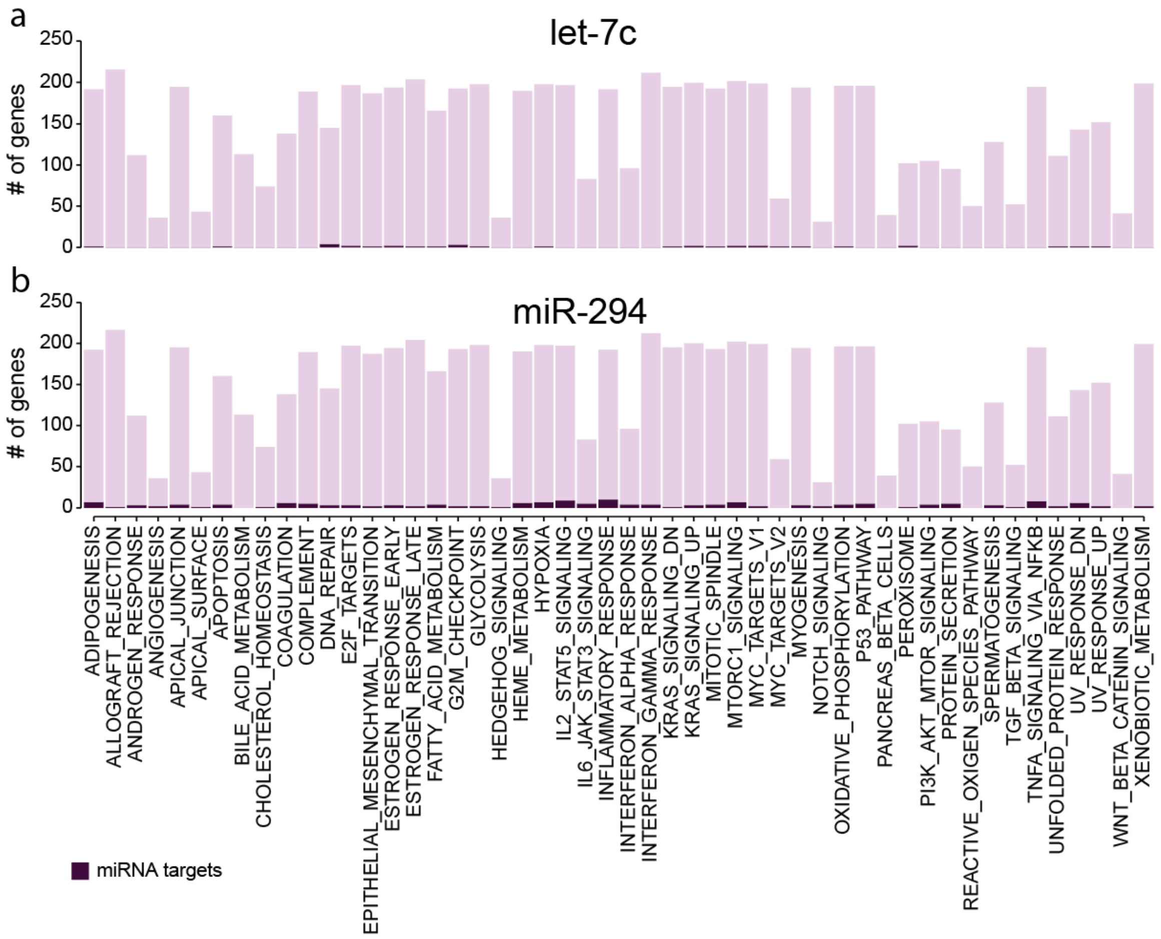

(**a**) Number of let-7c targets (dark violet) present in each one of the 50 MSigDb hallmarks gene sets. (**b**) Same as (a) but for miR-294 target genes.

Supplementary Figure 14: miR-294 and let-7c have opposing effects on transcriptional heterogeneity within the cell population

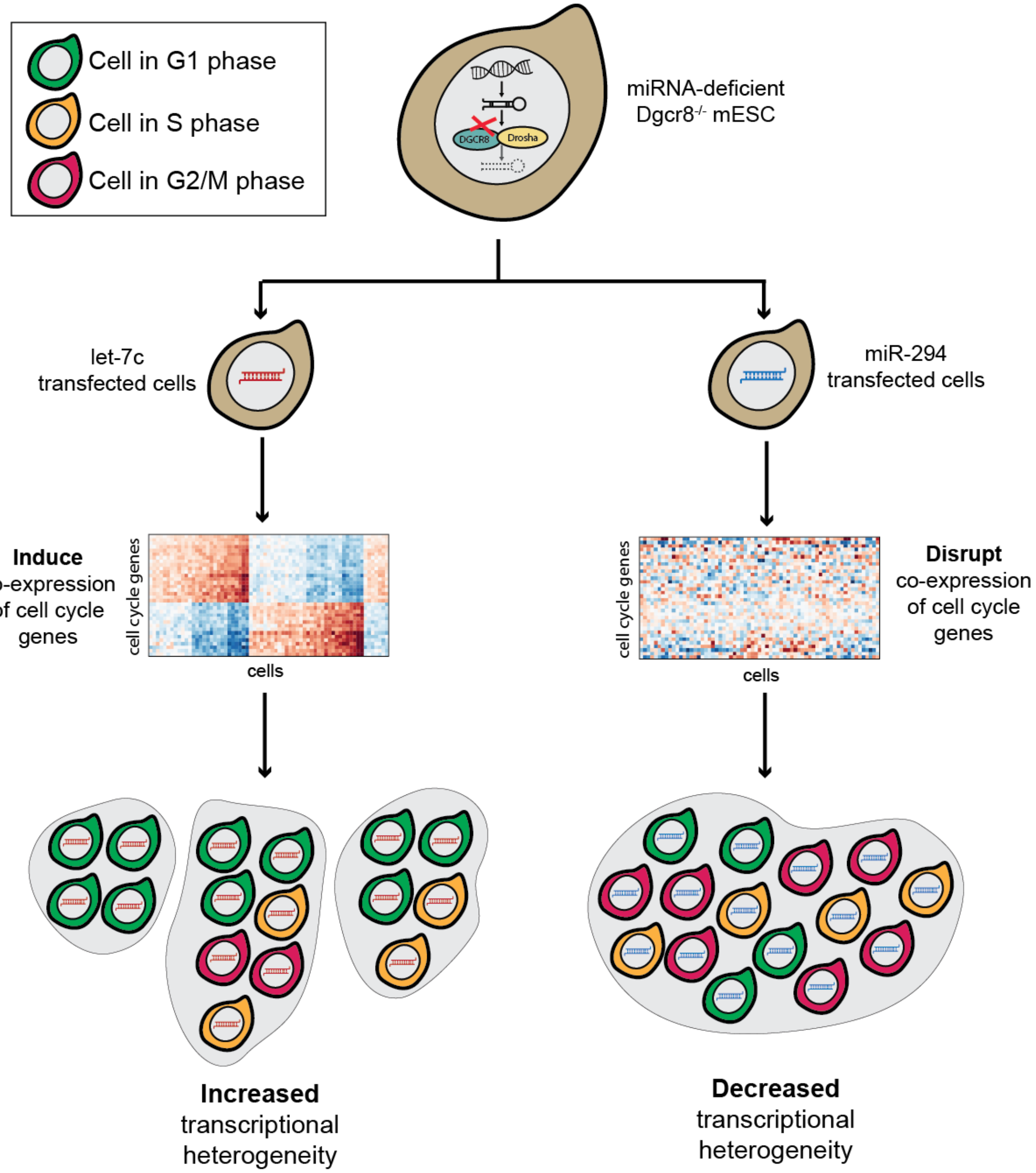

Let-7 induces co-expression of known cell cycle phase genes and increases transcription heterogeneity in ESCs. In contrast, miR-294 disrupts co-expression of the same genes while decreasing transcriptional heterogeneity within the cell population. The direct targets of the miRNAs that underlie this phenomenon remain to be determined.
